# The Release and Migration of Cr in the Soil under Alternating Wet–Dry Conditions

**DOI:** 10.3390/toxics12020140

**Published:** 2024-02-08

**Authors:** Zhe Chen, Ying Chen, Jing Liang, Zhiyu Sun, Haoren Zhao, Yi Huang

**Affiliations:** 1 State Key Laboratory of Geohazard Prevention and Geoenvironment Protection, College of Geosciences, Chengdu University of Technology, Chengdu 610059, China; chenzhe@stu.cdut.edu.cn (Z.C.); chenyinggeology@stu.cdut.edu.cn (Y.C.); romishin@163.com (H.Z.); 2 State Key Laboratory of Collaborative Control and Joint Remediation of Soil and Water Pollution, College of Ecology and Environment, Chengdu University of Technology, Chengdu 610059, China; 13036613815@163.com (J.L.); s1528680142@163.com (Z.S.)

**Keywords:** chromium, leaching, hexavalent chromium, sequential extraction

## Abstract

In recent decades, chromium contamination in soil has emerged as a serious environmental issue, demanding an exploration of chromium’s behavioral patterns in different soil conditions. This study aims to simulate the release, migration, and environmental impact of chromium (Cr) in contaminated soils under natural rainfall conditions (wet–dry cycles). Clean soils sourced from Panzhihua were used to cultivate chromium-containing soils. Simulated rainfall, prepared in the laboratory, was applied to the cultivated chromium-containing soils in indoor simulated leaching experiments. The experiments simulated three years of rainfall in Panzhihua. The results indicate that soils with higher initial Cr contents result in higher Cr concentrations in the leachate, but all soils exhibit a low cumulative Cr release. The leachate shows similar patterns in total organic carbon (TOC), pH, electrical conductivity, and Cr content changes. An analysis of the speciation of Cr in the soil after leaching reveals a significant decrease in the exchangeable fraction for each Cr species, while the residual and oxidizable Cr fractions exhibit notable increases. The wet–dry cycle has the following effects on the soil: it induces internal reduction reactions in the soil, leading to the reduction of Cr(VI) to Cr(III); it alters the binding of Cr ions to the soil, affecting the migration of chromium; and it involves microorganisms in chemical processes that consume organic matter in the soil. After three years of rainwater leaching, chromium-containing soils released a relatively low cumulative amount of total chromium, resulting in a reduced potential risk of groundwater system contamination. Most of the chromium in the chromium-containing soil is fixed within the soil, leading to less biotoxicity.

## 1. Introduction

The natural environment serves as a space for and the foundation for human existence, and soil plays a pivotal role within this environment. Soil is a vital reservoir for chemical elements in the atmosphere, hydrosphere, and biosphere. It forms the cornerstone of life activities for plants, animals, and microorganisms. However, soil is subject to various forms of pollution, with heavy metal contamination emerging as a critical issue within soil ecosystems [1,2,3].

Heavy metal pollutants in the soil environment tend to be readily adsorbed by soil colloids, leading to their long-term accumulation. These pollutants are also resistant to natural degradation, causing irreversible damage to soil quality. Chromium is a highly mobile heavy metal element within the transition metals group and is a potential toxic element in soil. In recent decades, chromium contamination in soil has become a severe environmental issue [4]. Hexavalent chromium (Cr(VI)) is considered one of the most hazardous pollutants in the environment and is classified as a Group 1 carcinogen by the International Agency for Research on Cancer (IARC). Due to its carcinogenic and toxic nature, it not only threatens crop yields and quality but also poses risks to human and animal health [5,6,7]. Therefore, studying the migration behavior of Cr in soil is of paramount importance.

The oxidation state of Cr plays a crucial role in its environmental behavior, with each valence state exhibiting different solubility, mobility, and toxicity characteristics. In the environment, chromium can exist in various valence states, including Cr(VI), Cr(V), Cr(IV), Cr(III), and Cr(0). The primary forms of chromium in soils and groundwater are Cr(III) and Cr(VI). In soil environments, soil pH is a critical parameter determining the speciation of Cr(VI) and its redox potential. When soil pH falls within the range of 0.7–6.5, Cr primarily exists as HCrO_4_^−^ and Cr_2_O_7_^2−^ anions. However, when soil pH exceeds 6.5, only the CrO_4_^2−^ ion remains [8,9]. Cr(VI) entering the soil system undergoes several transformations, including adsorption by soil colloids, reduction reactions with soil organic matter, and precipitation reactions with other soil components [10]. Fonseca et al. [11] investigated an adsorption model of chromium (Cr) in Portuguese soil under conditions with pH values of 2, 5, and 7. They found that the adsorption of Cr by soil decreased with an increase in pH. Jean-Soro et al. [12] conducted leaching experiments on Cr-contaminated soils using EDTA and citric acid, finding that EDTA leaching resulted in lower levels of Cr leaching from the soil compared to citric acid. Another study by Geng et al. [13] involved leaching experiments on tailings-area soils using different low-molecular-weight organic acids (oxalic acid, malic acid and citric acid), revealing that the sequence of Cr leaching from the soil was citric acid > oxalic acid > malic acid.

Acid rain is an environmental issue faced by countries worldwide. Acid rain leads to the acidification of water bodies and soil surfaces, causing significant environmental damage [14]. In light of this, researchers conduct indoor simulated rainfall experiments to study the leaching characteristics of heavy metal elements in soil under acid rain conditions [15,16,17,18,19]. Concerning soils subjected to simulated rainwater leaching, most researchers focus on changes in element speciation before and after leaching [20]. Wang et al. [21] conducted leaching experiments on lead–zinc tailings using simulated acid rain. They found that the concentration of Cr in the leachate was only 20 mg/L, which was lower compared to the concentrations of other heavy metals in the leachate. The majority of Cr was retained in the tailings. Yang et al. [22] conducted leaching experiments on acidic contaminated mine soil and found that during the leaching process, the concentration of Cr in the leachate fluctuated with changes in pH. When the pH exceeded 4.6, Cr(OH)_3_ began to generate. The soluble and insoluble Cr species also could have transformed among each other in the pH range of 4.6–8.5. Cederkvist et al. [23] conducted leaching experiments on soil using simulated rainfall with a neutral pH and elevated Cr concentrations under conditions simulating normal rainfall and extreme rainfall. The results indicated that under normal rainfall conditions, over 50% of Cr was retained by the soil, while under extreme rainfall conditions, approximately 20% of Cr was retained by the soil. Jin et al. [24] conducted leaching experiments on slag dumps generated from zinc-smelting activities using simulated acid rain with pH values of 3.3 and 5.5, as well as DI H_2_O. The results indicated that, compared to Cd, Cu, Pb, and Zn, Cr exhibited unique leaching behavior, with higher concentrations of Cr in the leachate during the later stages of the leaching events, contrary to other heavy metals.

Simulating rainfall under natural conditions should not only consider the issue of heavy metal leaching but also use a condition that closely resembles real scenarios—wet–dry alternation—as a crucial evaluation criterion. Under wet–dry alternation leaching patterns, the release of heavy metals in the soil and the speciation of heavy metals are influenced. Wet–dry alternation alters soil aeration and moisture content and, consequently, controls the effectiveness of heavy metal elements in the soil, as well as the content of soluble organic carbon (DOC) in the soil [25]. The structure and activity of microbial communities are also significantly affected by natural wet–dry alternations [26]. Microbes, under soil drought conditions, induce a stress response that leads to the synthesis of a large quantity of solutes to maintain osmotic balance inside and outside the cells [27], resulting in the consumption of substantial organic matter in the soil.

Panzhihua, located in southwestern China, is one of the four major iron-ore-mining areas in the country. The city is characterized by extensive mining activities and substantial ore extraction, resulting in the accumulation of a significant amount of tailings in various tailings dams. Heavy metal elements within these tailings gradually leach into the soil with rainwater, posing a potential threat to the soil environment [28,29,30,31]. With the advancement of industrialization, the discharge of a large amount of sulfides has made Panzhihua one of the most severely affected regions in Sichuan Province in terms of acid rain damage and soil heavy metal pollution. Therefore, this location is an ideal sampling site for conducting experiments on soil heavy metal pollution and acid rain leaching.

In summary, it is crucial to conduct research on the spatial distribution and migration mechanisms of heavy metal elements in soil. However, previous studies on the migration behavior and mechanisms of chromium elements under alternating wet and dry conditions have been limited. Furthermore, there has been a lack of research on the leaching characteristics of rainwater in soil systems already contaminated by Cr under simulated rainfall conditions with different pH levels, as well as the transformation of Cr during its migration. This study aims to simulate the release and migration of Cr in contaminated soils under natural rainfall conditions (wet–dry cycles) and its environmental implications.

## 2. Materials and Methods

### 2.1. Soil Sample Collection

Soil samples were collected in March 2019. Clean soil, obtained from an area approximately 4 km northwest of a tailings pond in Panzhihua, was used for subsequent experiments involving chromium-containing soil. The tools utilized for soil collection included wooden spoons, measuring tapes, and cloth bags, among others. Before sampling, the tools were wiped clean with paper to remove any dust or soil on their surfaces, ensuring that the collected samples would not contaminate each other. The collected soil samples were placed in prepared cloth bags, numbered, and air-dried naturally.

### 2.2. Column Leaching Experiment

This study employed laboratory-prepared simulated rainwater to conduct simulated leaching experiments indoors using clean soil from Panzhihua. The experiment simulated three years of rainfall in Panzhihua, with five rounds of leaching for each year. Following each leaching event, there was a one-day dry period. After completing the leaching for the first year, a dry period of seven days followed; then, the process continued for the second year’s leaching. Following the completion of the second year’s leaching, a dry period of 15 days was observed before conducting the final rainwater leaching for the third year.

#### 2.2.1. Soil Column Preparation

The soil column, peristaltic pump, and automatic collector are the three main components of the soil column device. The filling of the soil column first requires obtaining some basic parameters of the soil column device through calculations.

Soil Column: The soil column is constructed from organic glass, forming a cylinder measuring 12 cm in height and 3 cm in inner diameter and with a volume of 84.78 cm^3^. Both the upper and lower parts of the cylinder have organic glass bases with a hollow ring of the same size as the base, allowing for a tight connection between the column body and the base. The ring and the base have six corresponding screw holes evenly distributed along their circumferences, enabling the connection of the top and bottom bases with the hollow ring using screws. At the center of the base, there is a groove with a radius of 1.7 cm designed to hold a similarly sized filter which has several small holes and a 200-mesh filter membrane at the top to effectively prevent the spillage of fine soil particles. To facilitate the insertion of a 1.02 mm inner diameter percolation tube into the center of the filter at the bottom, a small hole is made at its lower part.

1. Peristaltic pump: The peristaltic pump is used to regulate the rate of liquid transfer.
2. Automatic collector: The automatic collector collects leachate.

**Figure 1:**
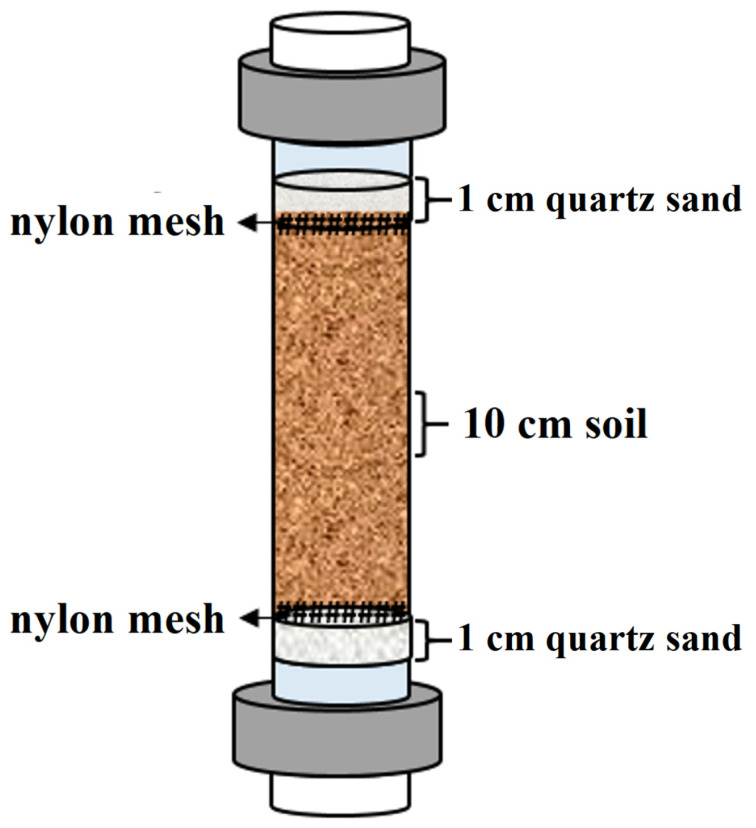
Schematic diagram of soil column.

Steps for filling the soil column are detailed in Text S1, and a simplified representation of the filled soil column can be found in Figure 1. After the soil column is filled, it requires the addition of a certain volume of deionized water from the base (0.4 times the weight of the soil inside the column) and saturation for 24 h to establish a chemical and biological balance within the soil column system. The mass of each soil column and other parameters used in the experiment can be found in Table 1.

#### 2.2.2. Contaminated Soil Preparation

For the contamination of soil containing 100 mg/kg of Cr, clean soil from Panzhihua was air-dried naturally for 15 days and sieved through a 10-mesh sieve. Then, 3 kg of soil was weighed using an electronic scale. The 3 kg of soil was placed in a 5 L beaker, and 750 mL of a potassium dichromate solution at a concentration of 100 mg/kg was added to the beaker. This resulted in soil with a moisture content of 25%. The soil was mixed thoroughly with the solution using a wooden spoon, and the beaker was then sealed with cling film. A certain number of small holes were made in the film with a needle for ventilation. The total mass of the beaker was recorded as M. During the incubation period, the soil was stirred once a day with a wooden spoon. Using a weighing method, water was added to keep the mass (M) of the beaker constant. This incubation process continued for 21 days. After incubation, the soil was removed and placed in a white cloth bag, air-dried naturally, sieved through a 10-mesh sieve, and set aside for further use.

When contaminating soil at rates of 200 mg/kg and 300 mg/kg of Cr, 750 mL amounts of potassium dichromate solutions with contents of 200 mg/kg and 300 mg/kg were separately added to the beakers. The remaining steps were the same as the method described above.

#### 2.2.3. Simulated Acid Rain Preparation

The reagents required to adjust the concentration of each component in simulated acid rain are of analytical purity and include the following:

CaCl_2_ (0.0005 mol/L), Ca(NO_3_)_2_ (0.0005 mol/L), MgCl_2_ (0.0005 mol/L), Na_2_SO_4_ (0.0001 mol/L), and KCl (0.0001 mol/L).

According to the data, the average precipitation in Panzhihua city in 2017 was 658 mm. The formula below was followed:

Simulated rainwater volume = Actual rainfall × Experimental column base area

We prepared 465 mL of rainwater intended to simulate the annual rainfall for this experiment. The pH of the rainwater was adjusted using solutions of HCl and KOH. This experiment aimed to simulate rainwater at three different pH values: 3, 5, and 7.

#### 2.2.4. Leaching Experiment

1. After filling the soil column, deionized water was introduced from the bottom, with a volume calculated as 40% of the weight of the soil inside the column. It was allowed to saturate for 24 h.
2. The pH of the rainwater was adjusted to 3 using solutions of HCl and KOH. The peristaltic pump flow rate was set to 1.55 mL/min. Ninety-three milliliters of this adjusted rainwater were introduced from the top into the soil column. The leachate was collected using the automatic collector, and the leaching process was allowed to complete. Afterward, the soil column was left to dry for 24 h. This process was repeated the following day and continued until a total of 465 mL of rainwater had been introduced (over 5 days). This completed the simulation of a year’s rainfall in the Panzhihua region. The soil column was allowed to dry for an additional 7 days.
3. After a 7-day dry period, the second year’s rainfall was simulated in the same manner, introducing a total of 465 mL of rainwater. After completing the leaching process, a drying period of 15 days was allowed.
4. After the 15-day dry period, the total rainfall for the third year was simulated in the same manner, introducing a total of 465 mL of rainwater. Upon completion of the leaching process, all leaching stages for this soil column were finished.
5. As the leaching process progressed, the pH, Eh, TOC, EC, Cr concentration, and Cr(VI) concentration of the leachate were continuously monitored.

#### 2.2.5. Sample Processing and Testing

After the soil column experiment was completed, the base of the soil column was dismantled. Soil samples were extracted using a sampler in 2 cm layers from the bottom to the top, resulting in a total of five layers of soil samples from the cross-section. The cross-sectional soil was packed into 50 mL centrifuge tubes and subjected to freeze-drying using a freeze dryer. During this process, the cross-sectional soil was placed in a freezing tray at −60 °C for 2 h; it was then transferred to a vacuum hood and dried for 8 h to complete the freeze-drying process. Subsequently, the cross-sectional soil was ground using an agate mortar, and finally, high-temperature and high-pressure closed-vessel digestion were employed to obtain the chromium element content. BCR sequential extraction was used to determine the fraction of chromium in the cross-sectional soil, while the determination of organic matter was conducted on soil samples from the middle layer (6 cm) of the soil profile.

### 2.3. Chromium Content

The content of Cr in the soil was determined by HNO_3_-HF high-pressure sealing digestion and ICP-MS. The concentration of Cr in the leachate was determined by ICP-MS. In order to ensure accuracy via the precision of the experiment, standard materials of the GSS series were used as standard samples. The determination value of each standard material was within a given range, and a blank sample was used as a zero reference for the instrument.

### 2.4. Sequential Extraction Experiment

In this paper, an improved BCR continuous extraction method was utilized to experimentally extract the fraction of heavy metal elements in the soil. Refer to Text S2 for specific steps.

### 2.5. Cr(Ⅵ) Determination

The determination of the Cr(VI) concentration referenced the national standard (GB7467-1987) and was measured using the diphenylcarbazide spectrophotometric method. Refer to Text S3 for specific steps.

### 2.6. Total Organic Carbon

A filtration process using a 0.45 μm filter membrane was employed on the leachate. Finally, the TOC (total organic carbon) value in the samples was measured using the TOC-L series total organic carbon analyzer by Shimadzu.

## 3. Results

### 3.1. Leachate

#### 3.1.1. Chromium Concentration

**Figure 2:**
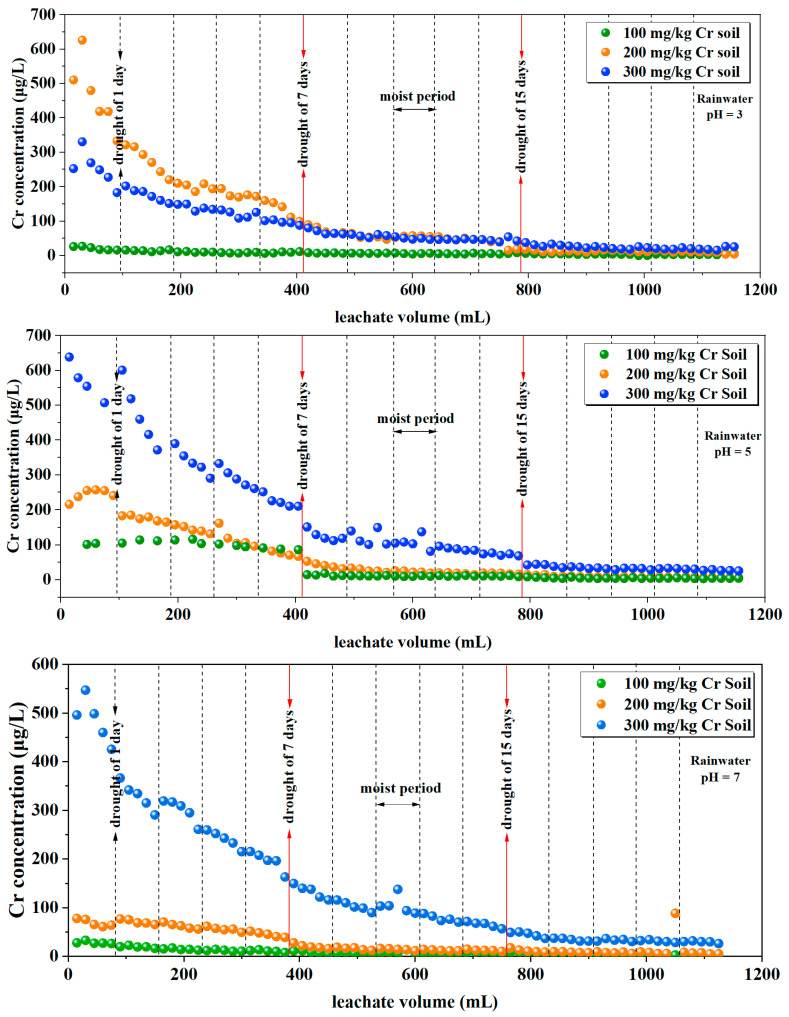
Variation in Cr concentration in the leachate.

The characteristics of the Cr content in the leachate are depicted in Figure 2. Initially, the overall trend observed in the variation in Cr content in the leachate indicates an increase corresponding to the initial addition of external Cr. The highest release of Cr occurs during the first year of simulated rainfall, followed by a decrease in the second year and a slow-release tendency in the third year, with released contents hovering around 100 ppb. For soil containing 100 mg/kg, 200 mg/kg, and 300 mg/kg of chromium, under rainwater with a pH of 3, the cumulative leached amounts of Cr are 0.6 mg, 8.6 mg, and 6.2 mg, respectively. Under rainwater with a pH of 5, the cumulative leached amounts are 1.8 mg, 5.1 mg, and 12.3 mg, respectively. With rainwater at a pH of 7, the cumulative leached amounts are 0.7 mg, 2.1 mg, and 11.0 mg, respectively.

Table 2 presents a statistical analysis of the leachate Cr concentration under different rainwater pH and different initial soil Cr content conditions. The results indicate that the leachate Cr concentration is significantly affected by rainwater pH (*p*-Value < 0.05) and highly significantly affected by the initial soil Cr content and the interaction of both factors (*p*-Value < 0.001).

#### 3.1.2. Redox Potential

**Figure 3:**
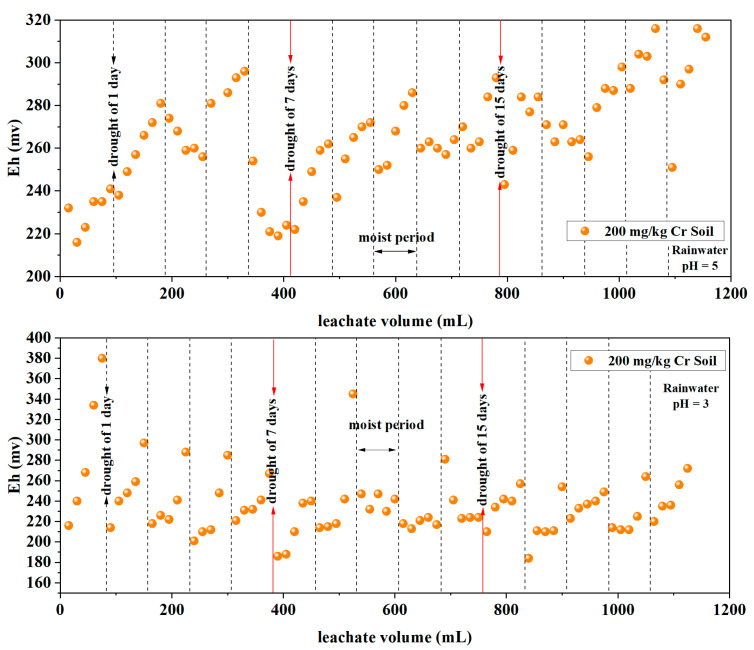

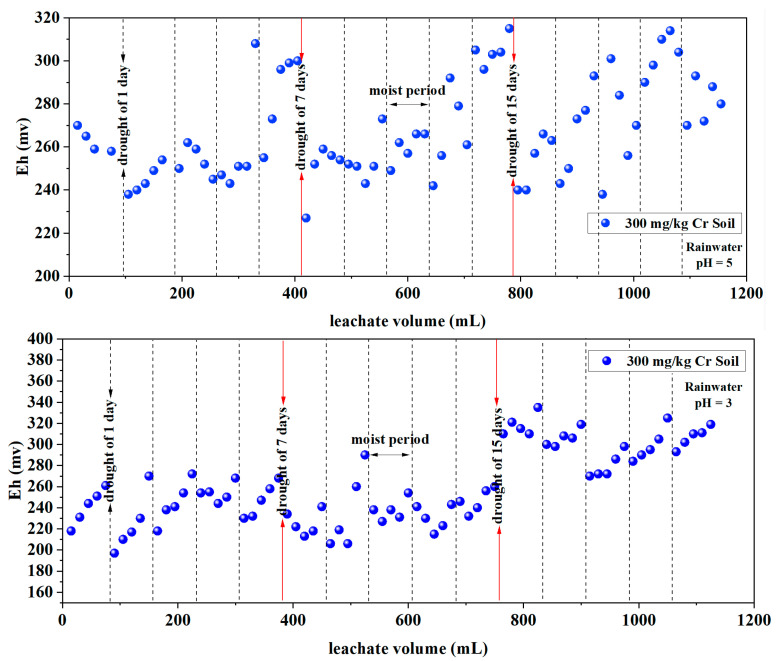
Variation in redox potential in the leachate.

One of the important indicators when measuring the strength of a solution’s oxidative and reductive properties is its redox potential (Eh). A higher Eh value indicates stronger oxidative conditions in a solution, while a lower Eh value corresponds to stronger reductive conditions. An Eh value > 0 signifies oxidizing conditions, whereas an Eh value < 0 indicates reducing conditions [32]. In soil, there are various redox systems such as oxygen, iron, manganese, nitrogen, sulfur, and organic systems. The speciation of different Cr ions in the soil is primarily influenced by factors like pH and Eh [33]. In this section, rainwater with pH values of 3 and 5 and soil Cr contents at 200 mg/kg and 300 mg/kg are selected for discussion, as shown in Figure 3.

The overall trend in the change in Eh values in the leachates from soils with two different initial contents (200 mg/kg and 300 mg/kg) is from low to high. For the leachate from the 200 mg/kg soil, at rainwater pH values of 3 and 5, the range of Eh values varied as follows: 184–380 mV and 216–329 mV, with average values of 237 mV and 266 mV, respectively. Regarding the leachate from the 300 mg/kg soil, at rainwater pH values of 3 and 5, the Eh value ranges were 197–335 mV and 227–315 mV, with average values of 260 mV and 267 mV, respectively. Table 3 presents a statistical analysis of leachate Eh under different rainwater pH and different initial soil Cr content conditions. The results indicate that leachate Eh is significantly affected by the initial soil Cr content (*p*-Value < 0.05) and highly significantly affected by rainwater pH and the interaction of both factors (*p*-Value < 0.001).

Under alternating wet–dry conditions, the change in Eh values in the leachate from each soil column showed a cyclic pattern, moving from low to high during leaching–drying–re-leaching cycles. This indicates that wet–dry cycles tend to lower the redox potential values in the soil leachate. Additionally, differences in this potential value were observed after 7 and 15 days of drought. The Eh values across all leachates ranged between 188 mV and 329 mV, depicting an overall oxidizing environment in the leachate.

#### 3.1.3. pH

**Figure 4:**
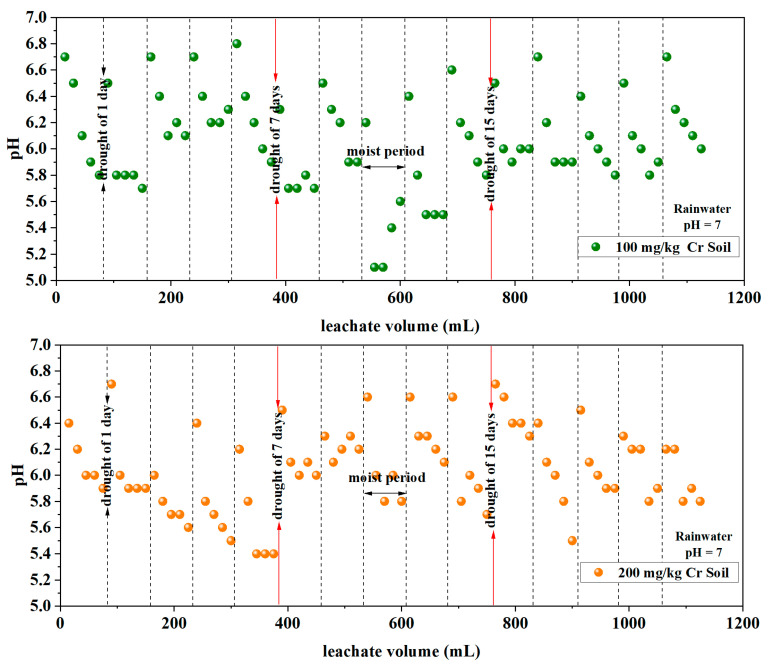

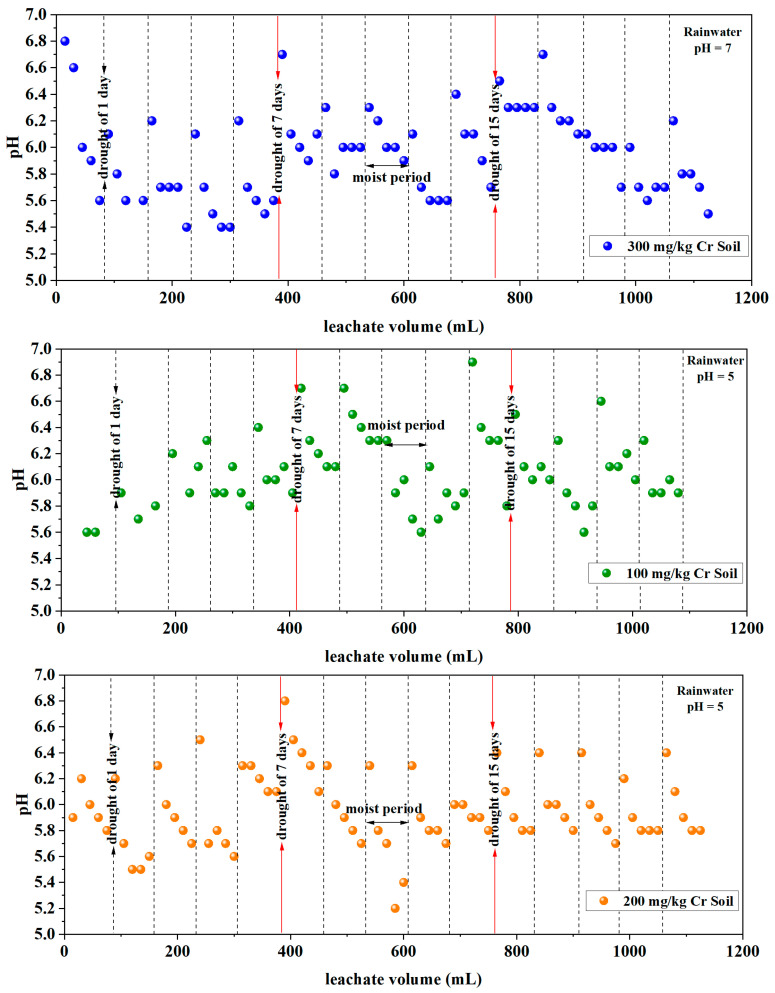

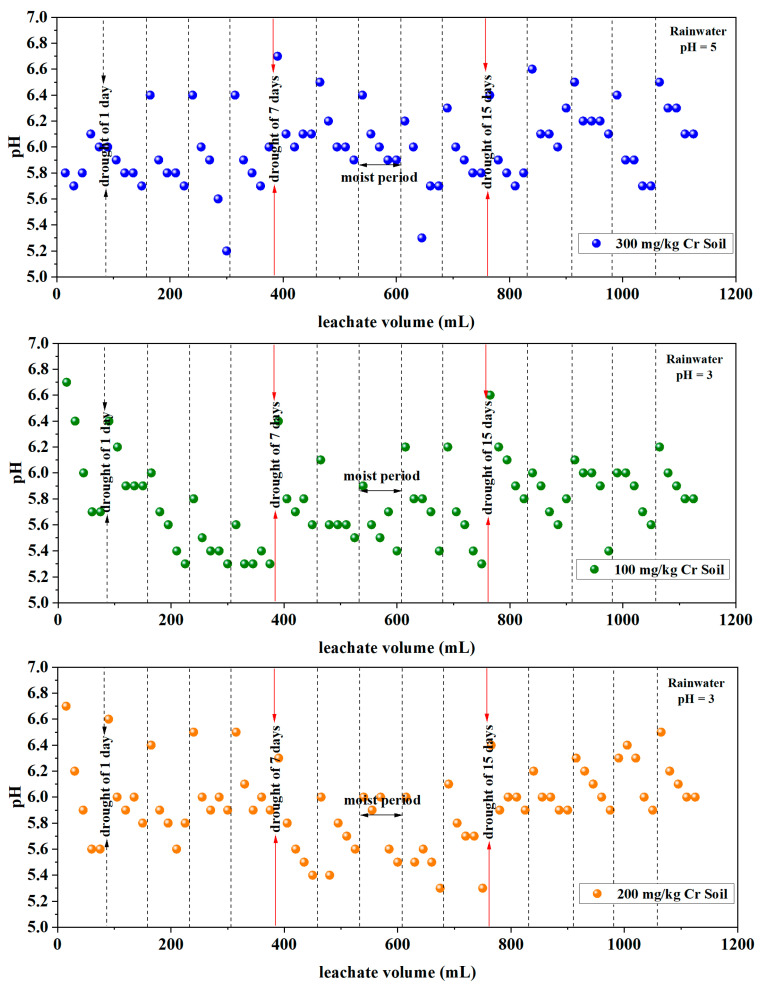

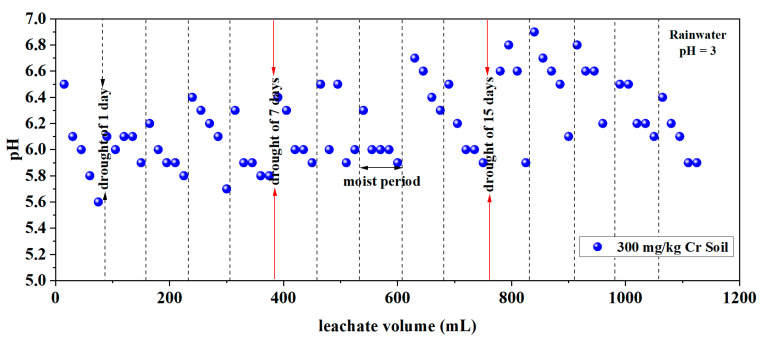
Variation in pH in the leachate.

The adsorption capacity of soil for heavy metals and the solubility of heavy metal insoluble salts are greatly influenced by the soil’s pH. When the solubility of salts in the soil changes, their mobility and biological activity correspondingly alter [34,35]. Under alternating wet–dry conditions of rainwater leaching, the pH variations in the leachate from soils with three different initial chromium contents under different rainwater pH conditions are depicted in Figure 4.

The pH variations in the leachate from each soil column ranged from 3.7 to 6.8, with average values ranging from 5.7 to 6.1, closely resembling the soil’s inherent pH (5.3). Even when rainwater with a pH of 3 was used, the pH in the leachate remained above 5. Table 4 presents a statistical analysis of leachate pH under different rainwater pH and different initial soil Cr content conditions. The results indicate that there is no significant correlation between rainwater pH and initial soil Cr content individually (*p*-Value > 0.05) with leachate pH. However, the combined effect of both factors significantly influences leachate pH (*p*-Value < 0.001). Similar to the patterns observed in the variations in Cr content and Eh values mentioned earlier, under alternating wet–dry leaching conditions, the pH in the soil leachate cyclically fluctuated from high to low during leaching–drying–re-leaching cycles.

#### 3.1.4. Electrical Conductivity

**Figure 5:**
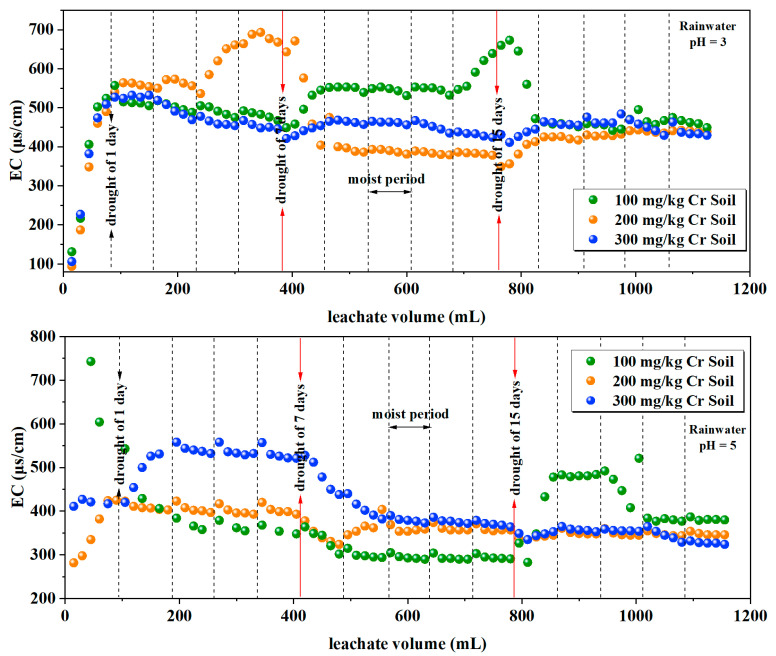

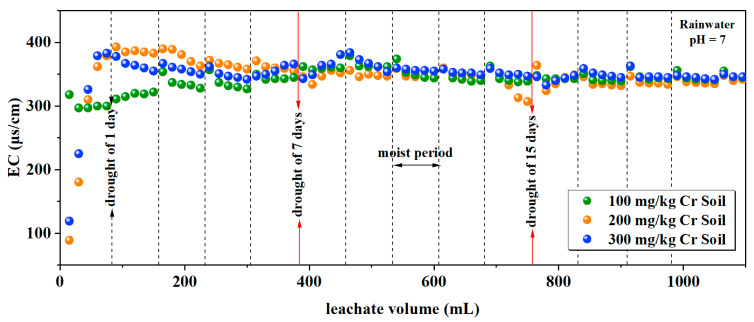
Variation in EC in the leachate.

Electrical conductivity (EC) is commonly used to indicate the leaching state of water-soluble ions in soil. Variations in electrical conductivity in the soil leachate are depicted in Figure 5. The ranges of electrical conductivity in the leachate at rainwater pH levels of 3, 5, and 7 were 93.8–693 μs/cm, 282–743 μs/cm, and 89–393 μs/cm, respectively. The respective average ranges of electrical conductivity were 451.3–501.5 μs/cm, 375.4–417.6 μs/cm, and 342–349.2 μs/cm. Table 5 presents a statistical analysis of leachate EC under different rainwater pH and different initial soil Cr content conditions. The results indicate that there is no significant correlation between leachate EC and initial soil Cr content (*p*-Value > 0.05). Leachate EC is significantly affected by rainwater pH and the combined interaction of both factors (*p*-Value < 0.001). The overall trend in electrical conductivity indicates that lower rainwater pH values lead to significantly higher conductivity values. The overall solubility of water-soluble ions in the leachate shows a decreasing trend, similar to the trend observed in Cr content. Under alternating wet–dry conditions, a 1-day interval of drought resulted in increased electrical conductivity upon re-leaching on the following day. However, extending the drought period, such as to 7 days or 15 days, resulted in lower conductivity values for most soil column leachates upon re-leaching.

#### 3.1.5. Total Organic Carbon

The concentration of carbon in water signifies the concentration of organic matter in the water, referred to as total organic carbon (TOC), expressed as a mass concentration of carbon (mg/L). Carbon is the core element constituting organic matter, and higher concentrations of organic matter in water lead to higher TOC values. Therefore, TOC values can be used to assess the extent of organic pollution in water.

**Figure 6:**
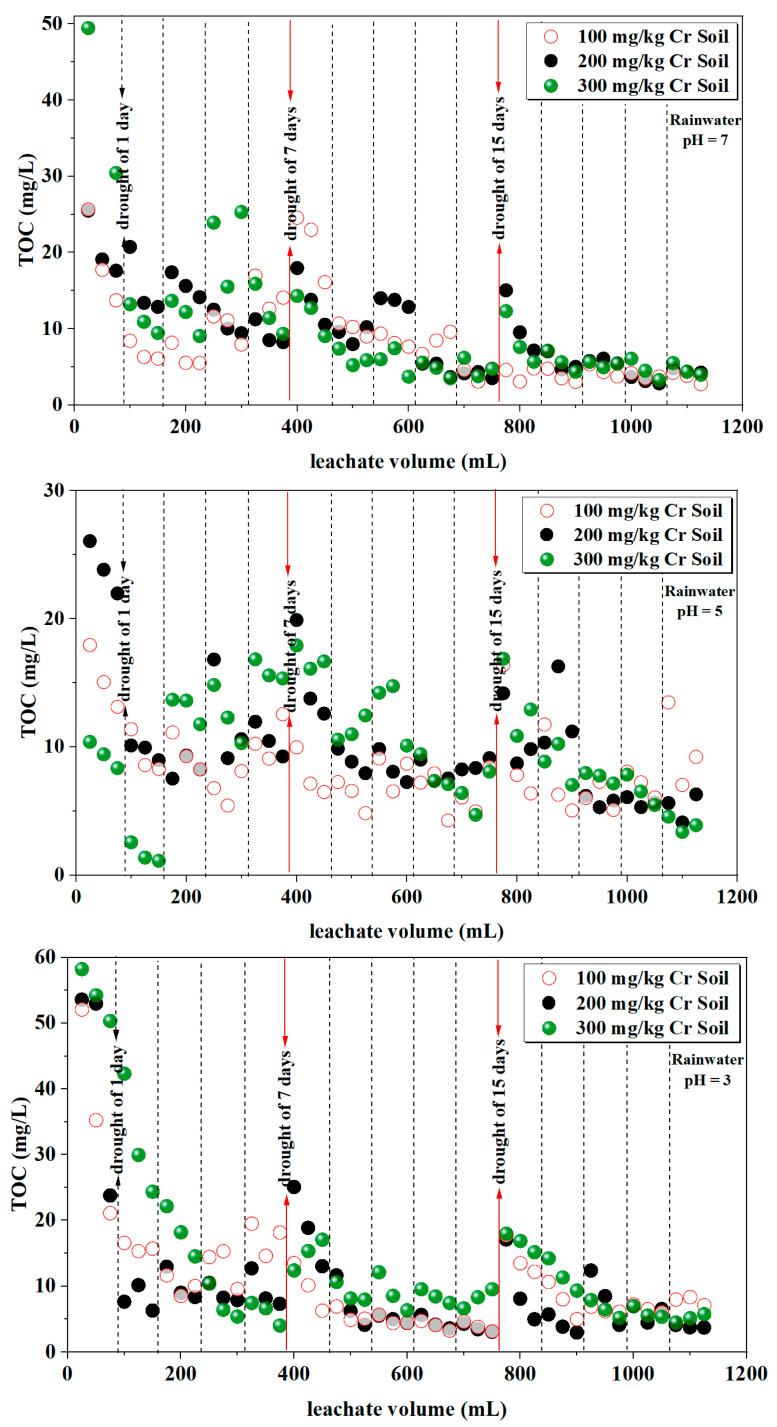
Variation in TOC in the leachate.

The total organic carbon content in the leachate is depicted in Figure 6. When filtering chromium-containing soil with rainwater of three different pH values, the TOC content in the leachate fluctuates between 1.1 mg/L and 58.2 mg/L. Table 6 presents a statistical analysis of leachate TOC under different rainwater pH and various initial soil Cr content conditions. The results suggest a slight correlation between leachate TOC and rainwater pH (*p*-Value = 0.048), with no significant correlation observed with the initial soil Cr content or their combined effects (*p*-Value > 0.05). The variations in TOC content across different columns of soil leachate are relatively similar, with average values ranging from 8.5 mg/L to 14.2 mg/L. The total TOC significantly decreases with an increase in leaching volume. Similar to trends observed in chromium content, electrical conductivity (EC), and pH, the wet–dry alternation notably increases the TOC content during moist periods.

#### 3.1.6. Cr(VI)

**Figure 7:**
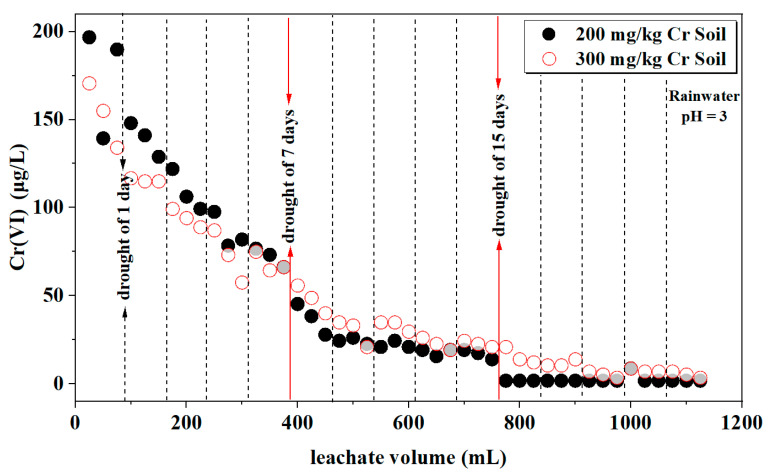

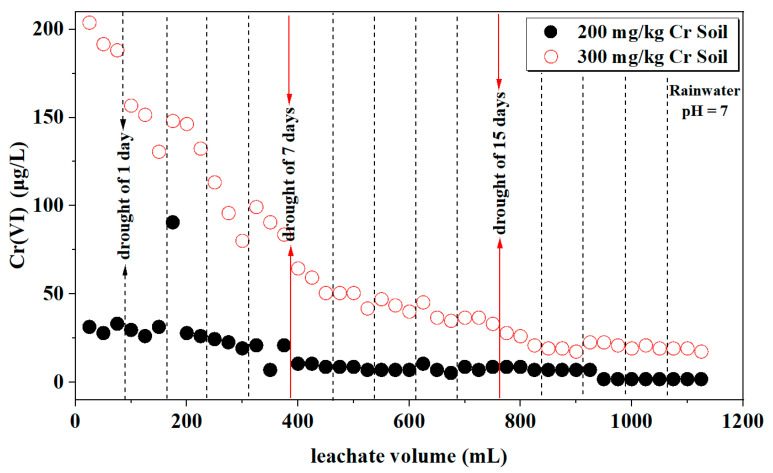
Variation in Cr(VI) concentration in the leachate.

Chromium in natural water bodies and soil solutions predominantly exists in two oxidation states: hexavalent and trivalent. Therefore, an analysis of Cr(VI) and Cr(III) in the soil leachate was necessary. In this section, rainwater samples with pH values of 3 and 7 and initial chromium contents of 200 and 300 mg/kg in the soil were chosen for discussion. Table 7 presents a statistical analysis of leachate Cr(VI) concentration under different rainwater pH and various initial soil Cr content conditions. The results indicate that there is no significant correlation between leachate Cr(VI) concentration and rainwater pH (*p*-Value > 0.05). Leachate Cr(VI) concentration is significantly affected by the initial soil Cr content and the combined interaction of both factors (*p*-Value < 0.001). As shown in Figure 7, when the rainwater pH is 3, the trends and release amounts of Cr(VI) in the leachates of the different soils are relatively consistent, ranging from 1 to 196 ppb. When the rainwater pH is 7, the combined effects of neutral rainwater and different initial soil Cr contents influence the release of Cr in the soil. Consequently, the corresponding release of Cr(VI) is affected, leading to a decrease in Cr(VI) release from the soil with an initial Cr content of 200 mg/kg.

### 3.2. Soil

#### 3.2.1. Chromium Content

**Figure 8:**
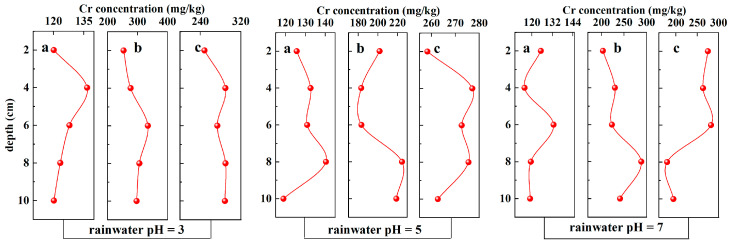
Cr contents in different layers of soil (a, b, c represent soils containing chromium at concentrations of 100 mg/kg, 200 mg/kg, and 300 mg/kg respectively).

The accumulation, toxicity, transformation, and migration of chromium in soil are closely associated with processes like adsorption and desorption at the soil interface [36]. The chromium contents in various soil layers of soils with different initial chromium contents after leaching with rainwater of varying pH values are depicted in Figure 8. Table 8 presents a statistical analysis of soil Cr content after leaching under different rainwater pH and different initial soil Cr content conditions. The results indicate that rainwater pH, initial soil Cr content, and their combined impact have a highly significant effect on soil Cr content after leaching (*p*-Value < 0.001).

It is evident that soils with higher initial chromium contents retain higher chromium contents after rainwater leaching. The chromium in each soil column primarily concentrates in the middle layer of the soil (6–8 cm), while the content in the shallow layers is notably lower.

#### 3.2.2. Sequential Extraction

**Figure 9:**
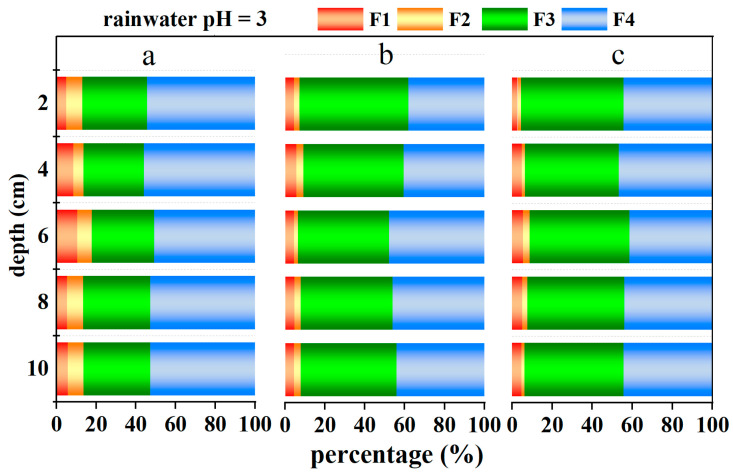

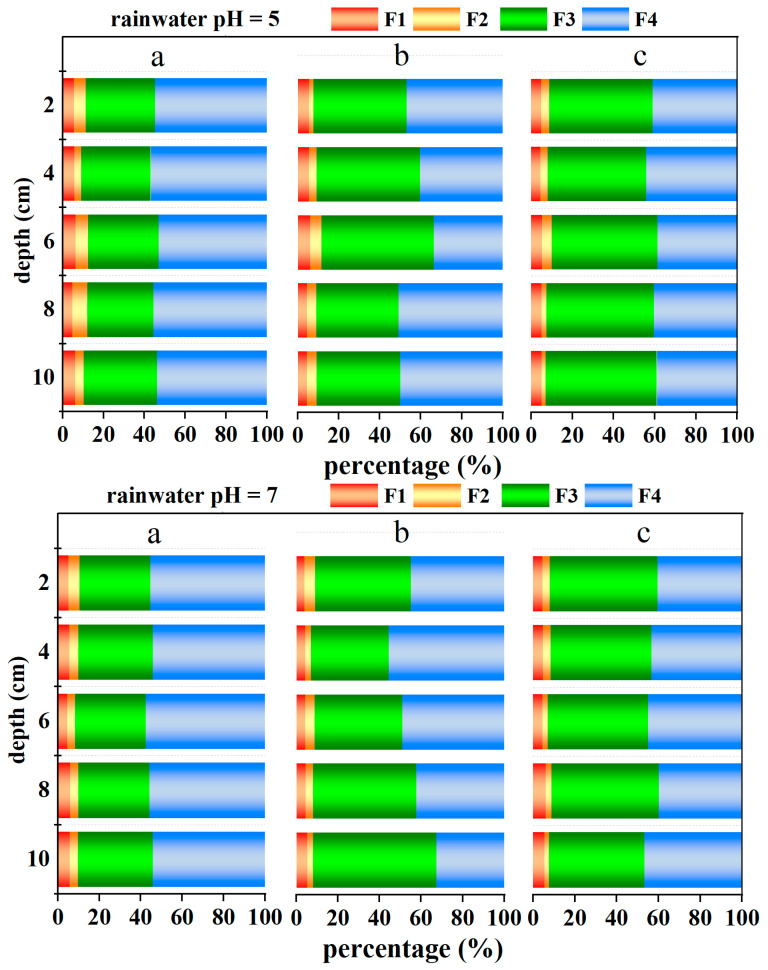
Chromium fraction characteristics in profiled soil (F1: exchangeable fraction, F2: reducible fraction, F3: oxidizable fraction, and F4: residual fraction; a, b, and c, respectively, represent soils with chromium contents of 100 mg/kg, 200 mg/kg, and 300 mg/kg).

The soil, post-simulated rainwater leaching, was extracted using the BCR sequential extraction method to acquire four fractions for each layer (2 cm) of soil. This was carried out to compare the characteristics of chromium in soils with three different initial contents before and after leaching. The distribution of chromium in various fractions is depicted in Figure 9. The chromium (Cr) fraction characteristics in chromium-containing soils, post rainwater leaching, generally indicate that after rainwater leaching, chromium primarily exists in an oxidizable fraction and residue fraction, with relatively lower proportions in exchangeable and reducible fractions.

Table 9 presents the statistical analysis of Cr content percentages in different soil fractions after leaching under different rainwater pH and different initial soil Cr content conditions. The results indicate that there is no significant correlation between rainwater pH and Cr content percentage after leaching for the four fractions (*p*-Value > 0.05); the soil’s initial Cr content is significantly correlated with the Cr content percentage after leaching in the exchangeable fraction (*p*-Value < 0.05). For the remaining three fractions, the soil’s initial Cr content is highly significantly correlated with the Cr content percentage after leaching (*p*-Value < 0.001); except for the highly significant impact of the combined effect of rainwater pH and initial soil Cr content on the percentage of Cr content in the reducible fraction (*p*-Value < 0.001), there are no significant correlations between the combined effect of rainwater pH and initial soil Cr content on the percentage of Cr content in the remaining three fractions (*p*-Value > 0.05).

#### 3.2.3. Total Organic Carbon

As an integral part of soil, organic matter plays a pivotal role in the soil’s structure and improving its physical conditions. A total organic carbon analysis was performed on the profiled soil after simulated rainfall leaching. The obtained organic carbon results were multiplied by an oxidation correction factor (1.724) to determine the organic matter content in the profiled soil. The findings are presented in Table 10.

The profiled soil samples from the mid-layer (6 cm) following leaching with rainfall at three different pH levels were compared with soil that had not undergone leaching to assess the organic matter content. According to Table 10, compared to the pre-leached soil’s organic matter content (30.85 mg/L), the organic matter content in the soil significantly decreased post leaching, measuring less than 1 mg/L. Post leaching, the organic matter content in the profiled soil ranged from 0.58 to 0.88 mg/L, showing no distinct correlation with the pH levels of rainfall (3, 5, and 7) or the initial Cr content in the soil (100, 200, and 300 mg/kg).

## 4. Discussion

### 4.1. Release of Cr from Soil to Leachate

The decreasing trend in chromium release with ongoing leaching according to Figure 2 could be attributed to the chromium salts (CrO_2_^4−^) that were added to the soil during the cultivation of the Cr-containing soil. Primarily, these enter the soil in a water-soluble form, with only a small portion being adsorbed by clay minerals in the soil [37]. This led to a rapid initial release of a significant amount of free CrO_2_^4−^ during the rainfall simulation, aligning with prior studies on the leaching characteristics of soil contaminated with external Se [19,38]. During the simulated rainfall leaching in the third year, a small amount of Cr continued to leach from each soil column. This could be due to competition between CrO_2_^4−^ and Cl^−^, NO_3_^−^, and SO_4_^2−^ in the simulated rainfall components for adsorption sites, resulting in the slow release of weakly bound Cr from the soil in the later stages of leaching. This aligns with the research findings of Li et al. [39] concerning As.

When individually examining each experimental group, after the soil column underwent leaching–drying–re-leaching, the Cr content increases compared to the previous leaching during the second re-leaching. This increase could be attributed to short-term wet–dry cycles that enhance the release of heavy metals in the soil. Changes in soil moisture during wet–dry cycles in rainfall leaching increase the content of water-soluble organic carbon (DOC), facilitating the migration of heavy metals [25]. Previous research has explored longer dry periods (45 days) in which, during the leaching–drying–re-leaching process, a significant release of Cr into the soil occurred [40]. This indicates that during drought, the valence state or speciation of Cr undergoes transformation. Some less-soluble forms of Cr convert to mobile forms and are quickly depleted. Simultaneously, the arid environment changes some soil and tailings voids from being filled with water to being filled with air, potentially converting some Cr(III) to CrO_2_^4−^, where Cr(VI) dominates in a strongly leachable form, easily migrating with water flow and thus increasing its leaching behavior. This leads to significant Cr release during the leaching–drying–re-leaching process.

When examining the release of Cr under three different rainwater pH conditions (Figure 2 and Figure 8), it was observed that as the rainwater pH decreases, the total content of Cr in each soil layer increases with a noticeable decrease in the release of Cr. Conversely, with a higher rainwater pH, the total content of Cr in each soil layer decreases, with a higher release of chromium. The highly significant correlation between rainwater pH and soil Cr content after leaching, as shown in Table 8, further confirms this observation (*p*-Value < 0.001). Table 2 also indicates that leachate Cr concentration is significantly influenced by rainwater pH (*p*-Value < 0.001). This suggests that in a lower-pH soil environment, more positive charges are present on the soil surface while CrO_2_^4−^ carries a negative charge, thereby resulting in increased adsorption of CrO_2_^4−^ onto the soil.

Additionally, based on Figure 8, after the leaching process, the majority of the Cr is primarily concentrated in the middle sections (6–8 cm) of the soil columns, whereas the content in the shallow layers is notably lower. However, experiments simulating V elements in acid rain leaching near smelters yielded different findings [16]: after ten years of rainwater leaching, the distribution of vanadium (V) elements in the soil was mainly concentrated in the surface layer (0–20 cm). Despite leaching with rainwater of varying pH values, the chromium content remained relatively high in the profiled soil, indicating that most of the chromium was fixed in the soil. This fixation might occur due to the encapsulation of Cr(III) by iron oxides or the formation of chromium–iron hydroxides, making it less mobile in the soil. Conversely, Cr(VI) is less prone to adsorption by the soil due to its anionic properties [41].

The variation in total organic carbon (TOC) in both the soil and leachate samples can similarly explain the release characteristics of Cr. From Table 6 and Figure 6, it can be observed that the correlation between leachate total organic carbon (TOC) and rainwater pH, as well as the initial soil Cr content, is not significant. Leachate TOC is mainly controlled by the conditions of wet–dry alternation. After a period of drought, there is a slight increase in the TOC content in the leachate, a phenomenon more pronounced after 7 and 15 days of drought. This suggests that with longer drought periods, there is a proportional increase in the released TOC content, likely due to heightened microbial respiration during the dry period, leading to the rapid release of unstable organic compounds from the soil. These compounds subsequently dissolve during subsequent wet periods [42]. Similar research has shown that during the early stages of acid rain leaching, a substantial amount of dissolved organic matter is released from the soil [16], providing a reasonable explanation for the variability in the TOC within the leachate. 

The variation in the organic matter content of the soil before and after simulated rainfall leaching was notably significant (Table 10). One possible reason is the set conditions of alternating wet and dry leaching in the soil column experiment. Soil microbes engaged in anaerobic respiration, leading to the consumption of a portion of the organic matter [43]. Another potential factor is related to humic substances within organic matter. Humic substances, stable, high-molecular-weight compounds formed during the decomposition or synthesis of organic matter, tend to complex with metal ions, aiding in eliminating excess metal ions in soil solutions. This complexation process might contribute to the depletion of organic matter, resulting in a reduction in Cr content in the soil.

During the adsorption process, organic matter and Cr compete for adsorption sites. A higher organic matter content in soil leads to a lower adsorption capacity for Cr, while soils with lower organic matter contents exhibit higher adsorption capacities for Cr. This explains why, in this study, after rainfall leaching, soils containing chromium had less accumulated Cr release in the leachate and more retained Cr content in the soil. 

### 4.2. Variation in Cr in Different Soil Fractions

The relatively lower contents of exchangeable and reducible fractions of Cr compared to oxidizable and residual fractions, as indicated in Figure 9, may be due to the substantial release of the water-soluble form of CrO_4_^2−^ from the soil during the initial leaching, leaving behind residual and oxidizable fractions of chromium in the soil.

The oxidizable fraction comprises various heavy metal–organic compound complexes within the soil, often reflecting aquatic biological activities and the discharge of organic-rich sewage by humans. Typically stable, the oxidizable fraction is usually released into the aquatic environment only under strong oxidative conditions. According to Table S1, higher initial contents of Cr in the soil profile correspond to higher proportions of the oxidizable fraction. The highly significant correlation between the initial soil Cr content and the percentage of Cr content in various fractions after leaching, as shown in Table 9, also confirms this point. Similar to the total amount of chromium, in most columns, the oxidizable fraction of chromium is notably higher in the mid-sections of the soil (4–8 cm). The influence of rainwater pH on the percentage of Cr content in various fractions of the soil after leaching is relatively limited (Table 9), and there is not a significant variation in the percentage of Cr content in various fractions of the soil after leaching under different rainwater pH conditions.

**Figure 10:**
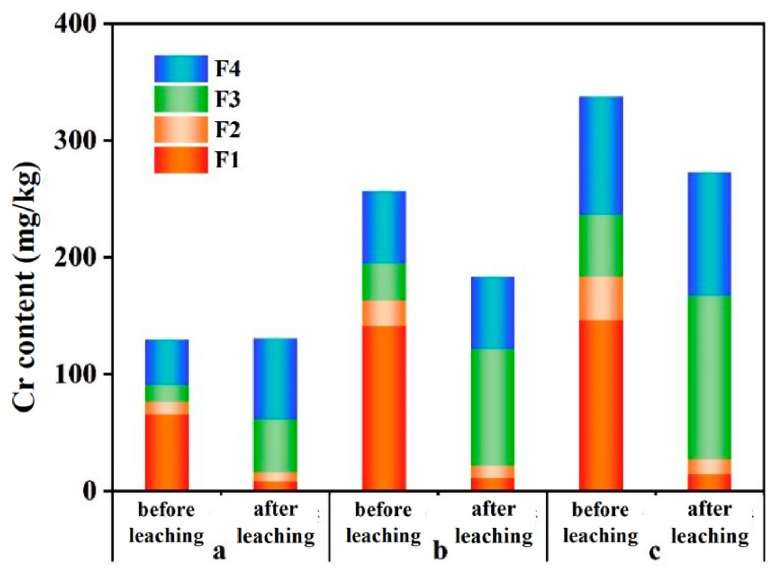
Chromium content distribution before and after leaching in chromium-containing soil fractions. (a, b, and c, respectively, represent soils with chromium contents of 100 mg/kg, 200 mg/kg, and 300 mg/kg).

Figure 10 illustrates the distribution of the Cr content in various fractions before and after leaching in soils with three different initial chromium contents. The pre-leaching chromium (Cr) content in the soil fractions was characterized by exchangeable fraction > residue fraction > oxidizable fraction > reducible fraction. Post rainwater simulation leaching, the Cr contents in various fractions of soils with three different initial chromium contents generally exhibited a decrease in the exchangeable fraction, a slight increase in the residue fraction, and an increase in the oxidizable fraction. Similar to this study, Mitchell et al. [44] also found a decrease in chromium content in the exchangeable fraction of the soil after leaching. This outcome demonstrates that CrO_4_^2−^, bound within soil particles, is readily released after prolonged rainwater leaching, posing an environmental hazard. The stability of the residue fraction in terms of content before and after leaching indicates that rainwater leaching over three years had minimal impact on altering the form of Cr within the soil lattice. Sarpong et al. [45] also found that compared to Cd and Mn, Cr in the soil remains predominantly in the oxidizable and residual fractions. However, the increased content of the oxidizable fraction after leaching suggests that a small portion of water-soluble CrO_4_^2−^ transformed into a more stable oxidizable fraction.

### 4.3. Redox of Cr under Alternating Wet–Dry Conditions

Based on the earlier discussion, it is evident that the Eh, pH, and conductivity in the leachate exhibited certain consistent patterns after leaching–drying–re-leaching. Here, we first discuss their characteristics separately.

As shown in Figure 4, under alternating wet–dry leaching conditions, the pH in the soil leachate cyclically fluctuated from high to low during leaching–drying–re-leaching cycles, but the pH in the leachate did not exhibit a significant correlation with the different initial contents of chromium in the soil (Table 4). This could be due to the soil’s buffering capacity, resulting in the pH of the leachate being above 5 when rainwater passes through it. During the initial stages of leaching, the soil’s basic ions undergo an ion exchange with H^+^ ions, alleviating the impact of simulated acid rain on the pH. However, as leaching progresses, the loss of basic ions from the soil increases, and the pH of the leachate is predominantly influenced by the simulated rainwater. This might be the reason for the fluctuation in pH from high to low observed during re-leaching.

According to Table 5, the leachate EC is highly correlated with rainwater pH, and the EC in the leachate is generally inversely related to the pH of the rainwater in the soil (Figure 5). Lower pH values of rainwater lead to a higher release of water-soluble ions in the leachate. This indicates that with an increase in leaching volume, the total quantity of ions in the solution decreases. The reason for this might be that various metal ions in the soil are relatively more soluble under acidic conditions. pH governs the way metals interact with the soil surface; H^+^ ions tend to replace metal cations bound to adsorption sites on the soil surface, thereby releasing various metal ions [19].

Based on Figure 3, the observed pattern of Eh values in the leachate, transitioning from low to high, might stem from redox reactions occurring within the soil column environment. The increase in soil moisture facilitates a reducing environment and the generation of Cr(III) in the soil. Therefore, after leaching–drying, internal redox reactions occur within the soil column. On the subsequent day of re-leaching, the Eh values tend to decrease compared to the previous day. Previous research indicates that when the redox potential value in the soil reaches several hundred millivolts, Cr_2_O_7_^2−^ will be reduced to insoluble trivalent chromium compounds [8]. Similar research [40] found that under alternating wet–dry conditions, a 45-day dry period did not significantly affect the variation in Eh values in the leachate when deionized water was used for leaching soil around mining areas. This might be attributed to an excessively prolonged dry period resulting in a complete loss of moisture throughout the soil profile, rendering the redox reactions in the soil inactive [46].

**Figure 11:**
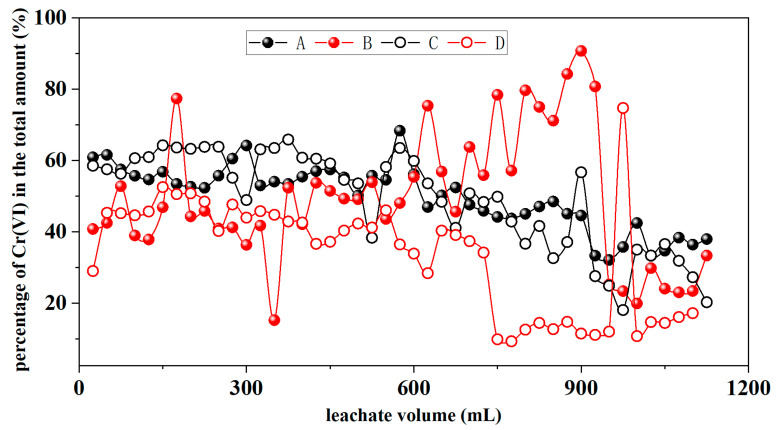
The proportion of Cr(VI) in the total amount of Cr in the leachate (A: pH = 7, soil with 300 mg/kg of chromium; B: pH = 7, soil with 200 mg/kg of chromium; C: pH = 3, soil with 300 mg/kg of chromium; D: pH = 3, soil with 200 mg/kg of chromium).

The observed changes in pH, EC, and Eh, combined with the characteristics of Cr(VI), suggest that redox reactions occurred within the soil column. Figure 11 illustrates the changes in the proportion of Cr(VI) in the leachate (specific concentrations of Cr(VI) and Cr(III) in the leachate can be found in Table S2). As shown in Figure 10 and Figure 11, it can be observed that there are substantial amounts of both Cr(VI) and Cr(III) in the leachate, and their proportions change daily. Overall, the trend shows an increasing proportion of Cr(III) with the progress of leaching, suggesting that a portion of Cr(VI) is being continuously reduced to Cr(III) during daily leaching. Moreover, when the rainwater pH is 7, the proportion of Cr(III) is higher in the later stages of leaching compared to a pH of 3. With an increase in the dry period (7 and 15 days), there is a significant decrease in the Cr(VI) content in the leachate compared to before the dry period, indicating that when all moisture is lost from the entire soil profile, the redox reactions in the soil become inactive.

Previous studies have shown that the redox reactions of chromium in soil are influenced by several factors, such as soil aeration, moisture content, the presence of Fe and Mn in the soil, microbial activity, organic matter content, and pH [47]. Additionally, organic matter exhibits strong redox capabilities, reducing the mobility and bioavailability of heavy metals. For example, Eckbo et al. [48] found a significant negative correlation between the concentration of Cr(VI) in leachate and the level of TOC, meaning that Cr(VI) leaching is reduced with an increasing TOC.

Based on the changes in pH, Eh, and Cr(VI) content, it can be inferred that under alternating wet and dry conditions [8], there will be changes in the soil column’s moisture content. Simultaneously, the soil’s aeration and organic matter content are likely to change, prompting redox reactions within the soil column [49]. Chromium’s migration behavior in the soil is primarily constrained by redox reactions and pH. The pH of the leachate decreases daily, while the Eh value increases, indicating that initially, Cr(VI) is being reduced to Cr(III).

## 5. Conclusions

This paper applied principles and methods from environmental geochemistry and trace element geochemistry. It conducted indoor soil column experiments using uncontaminated soil samples from the Panzhihua area, introducing external chromium pollution. The goal was to explore the migration behavior characteristics of chromium in contaminated soils under natural rainfall conditions. The study yielded the following conclusions and insights:

(1) Using indoor soil column experiments, simulated rainfall was applied to soils with different initial chromium contents, subjecting them to alternating wet and dry filtration cycles. The chromium concentration, pH, electrical conductivity, total organic carbon (TOC), and Cr(VI) content in the soil leachate exhibited similar changes. After filtration, drought, and re-filtration, there was a slight increase in the content of these indicators in the leachate, while the Eh value showed a slight decrease. Over three years of rainfall filtration, the soils containing chromium released a relatively small cumulative amount of chromium, minimizing potential pollution risks to the groundwater system.

(2) Analyzing soil samples before and after rainfall filtration revealed significant changes in their contents and forms: soils with higher initial chromium contents retained more chromium after rainfall filtration, with chromium concentrated in the middle layer (6–8 cm) of each soil column. Post- filtration, the overall trend in chromium’s elemental forms in the soil showed a significant decrease in the exchangeable fraction, a slight increase in the residual fraction, and a notable increase in the oxidizable fraction. After three years of rainfall filtration, a majority of the chromium in soils with chromium contamination was retained within the soil, resulting in less biological toxicity.

(3) After determining the oxidation states of chromium in the leachate, it was found that there was a significant presence of both Cr(VI) and Cr(III). The general trend observed was that with the progress of leaching, the proportion of Cr(III) increased, indicating a gradual reduction of Cr(VI) to Cr(III) during each daily leaching cycle. Based on the changes in Cr(VI) content, it was inferred that the soil columns’ moisture content, aeration, and organic matter altered during the cycles of wet and dry conditions, inducing redox reactions within the soil columns and influencing chromium’s migration behavior in the soil. The pH of the leachate decreased daily from high to low, while the Eh increased from low to high, indicating the initial reduction of Cr(VI) to Cr(III).

## Figures and Tables

**Table 1:** Different mass parameters of chromium-containing soil columns.

Rainwater pH	Soil Cr Content (mg/kg)	Soil Column Mass (g)	Soil Mass(g)	Distilled Water Volume (mL)
3	100	354.2	93.8	37.5
	200	354.2	97.6	39
	300	354.2	93	37.1
5	100	362.4	84.8	34
	200	361.0	86.4	34.4
	300	354.2	91.8	36.4
7	100	354.2	94	37.6
	200	354.2	96	38.4
	300	354.2	95	38

**Table 2:** Statistical analysis of leachate Cr concentration (μg/L) under different rainwater pH and different initial soil Cr content conditions.

		*n*		Mean		Std. Deviation		*p*-Value *	
Rainwater pH	3	224	664	8.02	73.43	98.69	107.71	0.014	<0.001
	5	241		115.31		122.75			
	7	225		83.56		99.23			
Initial Soil Cr Content	100 mg/kg	213	664	14.87	73.43	24.02	107.71	<0.001	
	200 mg/kg	227		70.22		96.68			
	300 mg/kg	224		132.37		133.02			

* *p*-Value, estimated using a two-way analysis of variance (ANOVA) and used to determine the significance of the individual and combined effects of initial soil Cr content and rainwater pH on the leachate Cr concentration.

**Table 3:** Statistical analysis of leachate Eh (mV) under different rainwater pH and different initial soil Cr content conditions.

		*n*		Mean		Std. Deviation		*p*-Value *	
Rainwater pH	3	225	663	245.07	255.7	33.58	33.14	<0.001	<0.001
	5	213		265.57		25.52			
	7	225		256.96		35.96			
Initial Soil Cr Content	100 mg/kg	214	663	253.76	255.7	32.82	33.14	0.048	
	200 mg/kg	225		253.23		34.60			
	300 mg/kg	224		260.02		31.62			

* *p*-Value, estimated using a two-way analysis of variance (ANOVA) and used to determine the significance of the individual and combined effects of initial soil Cr content and rainwater pH on leachate Eh.

**Table 4:** Statistical analysis of leachate pH under different rainwater pH and different initial soil Cr content conditions.

		*n*		Mean		Std. Deviation		*p*-Value *	
Rainwater pH	3	225	663	5.97	6	0.39	0.35	0.275	<0.001
	5	213		6.01		0.29			
	7	225		6.01		0.35			
Initial SoilCr Content	100 mg/kg	214	663	5.97	6	0.35	0.35	0.071	
	200 mg/kg	225		5.98		0.30			
	300 mg/kg	224		6.04		0.38			

* *p*-Value, estimated using a two-way analysis of variance (ANOVA) and used to determine the significance of the individual and combined effects of initial soil Cr content and rainwater pH on leachate pH.

**Table 5:** Statistical analysis of leachate EC (μs/cm) under different rainwater pH and different initial soil Cr content conditions.

		*n*		Mean		Std. Deviation		*p*-Value *	
Rainwater pH	3	225	663	472.08	401.77	87.22	86	<0.001	<0.001
	5	213		386.84		72.08			
	7	225		345.6		31.39			
Initial Soil Cr Content	100 mg/kg	214	663	395.74	401.77	99.31	86	0.173	
	200 mg/kg	225		403.27		85.32			
	300 mg/kg	224		406.03		71.9			

* *p*-Value, estimated using a two-way analysis of variance (ANOVA) and used to determine the significance of the individual and combined effects of initial soil Cr content and rainwater pH on leachate EC.

**Table 6:** Statistical analysis of leachate TOC (mg/L) under different rainwater pH and different initial soil Cr content conditions.

		*n*		Mean		Std. Deviation		*p*-Value *	
Rainwater pH	3	135	405	11.78	10.37	11.07	8.19	0.048	0.451
	5	135		9.57		4.23			
	7	135		9.76		7.68			
Initial Soil Cr Content	100 mg/kg	135	405	9.36	10.37	6.40	8.19	0.056	
	200 mg/kg	135		10.07		7.44			
	300 mg/kg	135		11.68		10.16			

* *p*-Value, estimated using a two-way analysis of variance (ANOVA) and used to determine the significance of the individual and combined effects of initial soil Cr content and rainwater pH on leachate TOC.

**Table 7:** Statistical analysis of leachate Cr(VI) concentration (μg/L) under different rainwater pH and different initial soil Cr content conditions.

		*n*		Mean		Std. Deviation		*p*-Value *	
Rainwater pH	3	90	180	47.22	43.52	49.87	48.73	0.273	<0.001
	7	90		39.82		47.55			
Initial Soil Cr Content	200 mg/kg	90	180	30.25	43.52	43.34	48.73	<0.001	
	300 mg/kg	90		56.79		50.41			

* *p*-Value, estimated using a two-way analysis of variance (ANOVA) and used to determine the significance of the individual and combined effects of initial soil Cr content and rainwater pH on leachate Cr(VI) concentration.

**Table 8:** Statistical analysis of soil Cr content(mg/kg) after leaching under different rainwater pH and different initial soil Cr content conditions.

		*n*		Mean		Std. Deviation		*p*-Value *	
Rainwater pH	3	15	45	232.39	210.74	80.73	69.18	<0.001	<0.001
	5	15		200.41		60.22			
	7	15		199.43		64.38			
Initial Soil Cr Content	100 mg/kg	15	45	125.90	210.74	7.36	69.18	<0.001	
	200 mg/kg	15		244.35		46.61			
	300 mg/kg	15		261.97		32.89			

* *p*-Value, estimated using a two-way analysis of variance (ANOVA) and used to determine the significance of the individual and combined effects of initial soil Cr content and rainwater pH on soil Cr content after leaching.

**Table 9:** Statistical analysis of Cr content percentage (%) in different soil fractions after leaching under different rainwater pH and different initial soil Cr content conditions.

			*n*		Mean		Std. Deviation		*p*-Value *	
F1	Rainwater pH	3	15	45	5.37	5.16	1.84	1.17	0.447	0.137
		5	15		5.21		0.61			
		7	15		4.91		0.67			
	Initial Soil Cr Content	100 mg/kg	15	45	5.96	5.16	1.56	1.17	0.003	
		200 mg/kg	15		4.74		0.60			
		300 mg/kg	15		4.79		0.72			
F2	Rainwater pH	3	15	45	4.15	4.03	2.51	1.75	0.203	<0.001
		5	15		4.31		1.48			
		7	15		3.63		0.92			
	Initial Soil Cr Content	100 mg/kg	15	45	5.62	4.03	1.76	1.75	<0.001	
		200 mg/kg	15		3.63		1.05			
		300 mg/kg	15		2.84		1.00			
F3	Rainwater pH	3	15	45	43.47	43.66	8.44	8	0.967	0.528
		5	15		43.85		8.19			
		7	15		43.65		7.92			
	Initial Soil Cr Content	100 mg/kg	15	45	33.86	43.66	1.64	8	<0.001	
		200 mg/kg	15		47.41		6.02			
		300 mg/kg	15		49.71		2.22			
F4	Rainwater pH	3	15	45	47.00	47.15	5.34	6.69	0.749	0.707
		5	15		46.64		7.46			
		7	15		47.81		7.46			
	Initial Soil Cr Content	100 mg/kg	15	45	54.57	47.15	1.76	6.69	<0.001	
		200 mg/kg	15		44.21		6.37			
		300 mg/kg	15		42.67		2.67			

F1—exchangeable fraction; F2—reducible fraction; F3—oxidizable fraction; F4—residual fraction. * *p*-Value, estimated using a two-way analysis of variance (ANOVA) and used to determine the significance of the individual and combined effects of initial soil Cr content and rainwater pH on Cr content percentage in different soil fractions after leaching.

**Table 10:** Content of organic matter in soil profiles.

Rainwater pH	Chromium-Containing Soil (mg/kg)	Organic Matter Content (mg/L)
Before Leaching		30.85
7	100	0.65
	200	0.88
	300	0.74
5	100	0.78
	200	0.79
	300	0.74
3	100	0.77
	200	0.58
	300	0.63

## Data Availability

Data are contained within the article and supplementary materials.

## Supplementary Materials

The following supporting information can be downloaded at https://www.mdpi.com/article/10.3390/toxics12020140/s1. Text S1: Method of filling the soil column; Text S2: Sequential extraction experiment; Text S3: Determination of Cr(Ⅵ) concentration; Table S1: Cr contents in various fractions of soil profile layers; Table S2. Concentrations and percentages of Cr(VI) and Cr(III) in the leachate.

## References

[1] Wang Y., Wang R., Fan L., Chen T., Bai Y., Yu Q., Liu Y. (2017). Assessment of multiple exposure to chemical elements and health risks among residents near Huodehong lead-zinc mining area in Yunnan, Southwest China. Chemosphere.

[2] Luo X., Yu L., Wang C., Yin X., Mosa A., Lv J., Sun H. (2017). Sorption of vanadium (V) onto natural soil colloids under various solution pH and ionic strength conditions. Chemosphere.

[3] Xia S., Song Z., Jeyakumar P., Bolan N., Wang H. (2020). Characteristics and applications of biochar for remediating Cr(VI)-contaminated soils and wastewater. Environ. Geochem. Health.

[4] Suzuki T., Kawai K., Moribe M., Niinae M. (2014). Recovery of Cr as Cr(III) from Cr(VI)-contaminated kaolinite clay by electrokinetics coupled with a permeable reactive barrier. J. Hazard. Mater..

[5] Kotaś J., Stasicka Z. (2000). Chromium occurrence in the environment and methods of its speciation. Environ. Pollut..

[6] Alvarez C.C., Bravo Gómez M.E., Hernández Zavala A. (2021). Hexavalent chromium: Regulation and health effects. J. Trace Elem. Med. Biol..

[7] Sharma P., Singh S.P., Parakh S.K., Tong Y.W. (2022). Health hazards of hexavalent chromium (Cr (VI)) and its microbial reduction. Bioengineered.

[8] Huang L., Yu C., Hopke P., Lioy P., Buckley B., Shin J., Fan Z. (2014). Measurement of Soluble and Total Hexavalent Chromium in the Ambient Airborne Particles in New Jersey. Aerosol Air Qual. Res..

[9] Wani K.I., Naeem M., Aftab T. (2022). Chromium in plant-soil nexus: Speciation, uptake, transport and sustainable remediation techniques. Environ. Pollut..

[10] de Souza Braz A.M., Fernandes A.R., Ferreira J.R., Alleoni L.R.F. (2013). Distribution coefficients of potentially toxic elements in soils from the eastern Amazon. Environ. Sci. Pollut. Res..

[11] Fonseca B., Teixeira A., Figueiredo H., Tavares T. (2009). Modelling of the Cr(VI) transport in typical soils of the North of Portugal. J. Hazard. Mater..

[12] Jean-Soro L., Bordas F., Bollinger J.C. (2012). Column leaching of chromium and nickel from a contaminated soil using EDTA and citric acid. Environ. Pollut..

[13] Geng H., Wang F., Yan C., Tian Z., Chen H., Zhou B., Yuan R., Yao J. (2020). Leaching behavior of metals from iron tailings under varying pH and low-molecular-weight organic acids. J. Hazard. Mater..

[14] Zhang Y., Li J., Tan J., Li W., Singh B.P., Yang X., Bolan N., Chen X., Xu S., Bao Y. (2023). An overview of the direct and indirect effects of acid rain on plants: Relationships among acid rain, soil, microorganisms, and plants. Sci. Total Environ..

[15] Zheng S., Zheng X., Chen C. (2012). Leaching Behavior of Heavy Metals and Transformation of Their Speciation in Polluted Soil Receiving Simulated Acid Rain. PLoS ONE.

[16] Xiao X., Jiang Z., Guo Z., Wang M., Zhu H., Han X. (2017). Effect of simulated acid rain on leaching and transformation of vanadium in paddy soils from stone coal smelting area. Process. Saf. Environ. Prot..

[17] Huang B., Li Z., Huang J., Chen G., Nie X., Ma W., Yao H., Zhen J., Zeng G. (2015). Aging effect on the leaching behavior of heavy metals (Cu, Zn, and Cd) in red paddy soil. Environ. Sci. Pollut. Res..

[18] Li J., Jia C., Lu Y., Tang S., Shim H. (2015). Multivariate analysis of heavy metal leaching from urban soils following simulated acid rain. Microchem. J..

[19] Zhai H., Xue M., Du Z., Wang D., Zhou F., Feng P., Liang D. (2019). Leaching behaviors and chemical fraction distribution of exogenous selenium in three agricultural soils through simulated rainfall. Ecotoxicol. Environ. Saf..

[20] Wang P., Sun Z., Hu Y., Cheng H. (2019). Leaching of heavy metals from abandoned mine tailings brought by precipitation and the associated environmental impact. Sci. Total Environ..

[21] Wang H., Ju C., Zhou M., Chen J., Kan X., Dong Y., Hou H. (2022). Acid rain-dependent detailed leaching characteristics and simultaneous immobilization of Pb, Zn, Cr, and Cd from hazardous lead-zinc tailing. Environ. Pollut..

[22] Yang L., Wei T., Li S., Lv Y., Miki T., Yang L., Nagasaka T. (2021). Immobilization persistence of Cu, Cr, Pb, Zn ions by the addition of steel slag in acidic contaminated mine soil. Hazard. Mater..

[23] Cederkvist K., Ingvertsen S.T., Jensen M.B., Holm P.E. (2013). Behaviour of chromium(VI) in stormwater soil infiltration systems. Appl. Geochem..

[24] Jin Z., Liu T., Yang Y., Jackson D. (2014). Leaching of cadmium, chromium, copper, lead, and zinc from two slag dumps with different environmental exposure periods under dynamic acidic condition. Ecotoxicol. Environ. Saf..

[25] Li Z., Wu L., Zhang H., Luo Y., Christie P. (2015). Effects of soil drying and wetting-drying cycles on the availability of heavy metals and their relationship to dissolved organic matter. J. Soils Sediments.

[26] Pang X., Chen C., Sun J., Zhan H., Xiao Y., Cai J., Yu X., Liu Y., Long L., Yang G. (2023). Effects of complex pollution by microplastics and heavy metals on soil physicochemical properties and microbial communities under alternate wetting and drying conditions. J. Hazard. Mater..

[27] Schimel J., Balser T.C., Wallenstein M. (2007). Microbial stress-response physiology and its implications for ecosystem function. Ecology.

[28] Barsanti M., Garcia-Tenorio R., Schirone A., Rozmaric M., Ruiz-Fernández A.C., Sanchez-Cabeza J.A., Delbono I., Conte F., Godoy J.M.D.O., Heijnis H. (2020). Challenges and limitations of the 210Pb sediment dating method: Results from an IAEA modelling interlaboratory comparison exercise. Quat. Geochronol..

[29] Cheng Q., Lou G., Huang W., Li X. (2017). Assessment and potential sources of metals in the surface sediments of the Yellow River Delta, Eastern China. Environ. Sci. Pollut. Res..

[30] Christophoridis C., Dedepsidis D., Fytianos K. (2009). Occurrence and distribution of selected heavy metals in the surface sediments of Thermaikos Gulf, N. Greece. Assessment using pollution indicators. J. Hazard. Mater..

[31] Rodríguez L., Ruiz E., Alonso-Azcárate J., Rincón J. (2009). Heavy metal distribution and chemical speciation in tailings and soils around a Pb–Zn mine in Spain. J. Environ. Manag..

[32] Bohn H.L. (1971). Redox Potentials. Soil Sci..

[33] Frohne T., Diaz-Bone R.A., Du Laing G., Rinklebe J. (2015). Impact of systematic change of redox potential on the leaching of Ba, Cr, Sr, and V from a riverine soil into water. J. Soils Sediments.

[34] Kicińska A., Pomykała R., Izquierdo-Díaz M. (2021). Changes in soil pH and mobility of heavy metals in contaminated soils. Eur. J. Soil Sci..

[35] Naz M., Dai Z., Hussain S., Tariq M., Danish S., Khan I.U., Qi S., Du D. (2022). The soil pH and heavy metals revealed their impact on soil microbial community. J. Environ. Manag..

[36] Zhang X., Tong J., Hu B., Wei W. (2018). Adsorption and desorption for dynamics transport of hexavalent chromium (Cr(VI)) in soil column. Environ. Sci. Pollut. Res..

[37] Yu J., Klarup D. (1994). Extraction kinetics of copper, zinc, iron, and manganese from contaminated sediment using disodium ethylenediaminetetraacetate. Water Air Soil Pollut..

[38] Dhillon S.K., Dhillon K.S., Kohli A., Khera K.L. (2008). Evaluation of leaching and runoff losses of selenium from seleniferous soils through simulated rainfall. J. Plant Nutr. Soil Sci..

[39] Li J., Kosugi T., Riya S., Hashimoto Y., Hou H., Terada A., Hosomi M. (2018). Pollution potential leaching index as a tool to assess water leaching risk of arsenic in excavated urban soils. Ecotoxicol. Environ. Saf..

[40] Yang J., Tang Y., Yang K., Rouff A., Elzinga E., Huang J. (2014). Leaching characteristics of vanadium in mine tailings and soils near a vanadium titanomagnetite mining site. J. Hazard. Mater..

[41] Fendorf S.E. (1995). Surface Reactions of Chromium in Soils and Waters. Geoderma.

[42] Merckx R., Brans K., Smolders E. (2001). Decomposition of dissolved organic carbon after soil drying and rewetting as an indicator of metal toxicity in soils. Soil Biol. Biochem..

[43] Conesa H.M., María-Cervantes A., Álvarez-Rogel J., González-Alcaraz M.N. (2011). Influence of soil properties on trace element availability and plant accumulation in a Mediterranean salt marsh polluted by mining wastes: Implications for phytomanagement. Sci. Total Environ..

[44] Mitchell K., Trakal L., Sillerova H., Avelar-González F.J., Guerrero-Barrera A.L., Hough R., Beesley L. (2018). Mobility of As, Cr and Cu in a contaminated grassland soil in response to diverse organic amendments; a sequential column leaching experiment. Appl. Geochem..

[45] Sarpong L., Boadi N.O., Akoto O. (2023). Metal Fractionation and Leaching in Soils from a Gold Mining Area in the Equatorial Rainforest Zone. J. Chem..

[46] Masscheleyn P.H., Pardue J.H., DeLaune R.D., Patrick J.W. (1992). Chromium redox chemistry in a Lower Mississippi Valley bottomland hardwood wetland. Environ. Sci. Technol..

[47] Banks M.K., Schwab A.P., Henderson C. (2006). Leaching and reduction of chromium in soil as affected by soil organic content and plants. Chemosphere.

[48] Eckbo C., Okkenhaug G., Hale S.E. (2022). The effects of soil organic matter on leaching of hexavalent chromium from concrete waste: Batch and column experiments. J. Environ. Manag..

[49] Lakatos J., Brown S.D., Snape C.E. (2002). Coals as sorbents for the removal and reduction of hexavalent chromium from aqueous waste streams. Fuel.

