# Supplementary material for: The Release and Migration of Cr in the Soil under Alternating Wet–Dry Conditions
Source: Toxics. 2024 Feb 8;12(2):140. doi: 10.3390/toxics12020140 (PMC10891877; doi:10.3390/toxics12020140)
Supplement: Supplementary file 1 [file toxics-12-00140-s001.zip › toxics-2830760-supplementary.pdf]

toxics

MDPI

Supplementary materials

The Release and Migration of Cr in the Soil under Alternating Wet–Dry Conditions

Zhe Chen [1], Ying Chen [1], Jing Liang [2], Zhiyu Sun [2], Haoren Zhao [1] and Yi Huang [1,2,*]

[1] State Key Laboratory of Geohazard Prevention and Geoenvironment Protection, College of Geosciences, Chengdu University of Technology, Chengdu 610059, China; chenzhe@stu.cdut.edu.cn (Z.C.); chenyinggeology@stu.cdut.edu.cn (Y.C.); romishin@163.com (H.Z.)

[2] State Key Laboratory of Collaborative Control and Joint Remediation of Soil and Water Pollution, College of Ecology and Environment, Chengdu University of Technology, Chengdu 610059, China; 13036613815@163.com (J.L.); s1528680142@163.com (Z.S.)

* Correspondence: huangyi@cdut.edu.cn

3. Texts and 2 Tables

Text S1 Method of Filling the Soil Column

The method of filling the soil column follows these steps:

(1) Begin by placing the filter at the center groove of the base of the soil column. At the top of the filter, put a 200-mesh filter membrane of the same size. Apply Vaseline uniformly to the connection between the lower base of the soil column and the hollow ring, as well as the inner wall, to prevent moisture from the soil column from overflowing. Connect them using screws to form an integrated base unit. Insert the soil column body into the integrated base unit. Repeat the previous operation at the top. Finally, seal the top and bottom percolation holes with plastic plugs of the same diameter. Remove the integrated base from the top of the soil column, secure the complete soil column unit using an iron frame stand, and position it vertically on the tabletop.

(2) Slowly add cleaned quartz sand into the soil column using a small spoon until the thickness of the quartz sand inside the column reaches 1cm, ensuring even percolation of rainwater. Place a pre-cut 200-mesh nylon mesh as an isolation layer to prevent soil and quartz sand from leaching into each other, gently spreading it over the surface of the quartz sand. Slowly add an equal amount of soil into the column using a small spoon according to the soil bulk density, trying to avoid soil contact with the inner wall. After filling each 1cm layer of soil, use a rubber hammer to evenly tamp the outer wall of the soil column to ensure uniform soil packing. Record the mass of the soil filled when the soil thickness reaches 10 cm. Repeat the previous steps by adding the 200-mesh nylon mesh film and quartz sand accordingly.

(3) Apply Vaseline evenly on the inner wall and junction of the top base. Then, screw this base into the upper end of the soil column and seal the percolation hole in the center of the top base using a plastic plug of the corresponding diameter.

Text S2 Sequential Extraction Experiment

exchangeable fraction

Add 20 ml of 0.11 mol/L acetic acid to a centrifuge tube, tightly seal the tube, and place it on a constant temperature oscillator at 220 rpm for 16 hours at room temperature to ensure thorough contact between the sample and the acetic acid. After agitation, centrifuge the tube at 3600 rpm for 15 minutes. Extract 9 ml of the supernatant using a 0.45 μm filter and transfer it to a 10 ml centrifuge tube, then add 1 ml of 20% nitric acid and discard the excess supernatant. Finally, wash the residue twice with 10 ml of ultrapure water.

reducible fraction

Add 20 ml of 0.5 mol/L hydroxylamine hydrochloride solution to the residue from step (1). Seal the centrifuge tube tightly and oscillate it at 22±5°C for 16 hours on an oscillator. The subsequent cleaning and transfer processes are the same as in step (1).

oxidizable fraction

Slowly add 5 ml of 30% H_2O_2 to the residue from step (2). Loosely twist the centrifuge tube cap to close it but not tightly to prevent gas blockage and potential sample loss due to tube bursting. Let the sample react with the solution at room temperature for 1 hour. Then transfer it to a water bath at 85±2°C and heat for about 1 hour. Remove the cap and continue heating at this temperature until the solution is nearly dry (approximately 2-3 ml remaining). Add 5 ml of 30% H_2O_2 (pH 2-3), loosely twist the centrifuge tube cap, and heat in the water bath at 85±2°C for approximately 1 hour. Remove the cap, and evaporate the sample at 85±2°C until approximately 2-3 ml of solution remains. Take out the centrifuge tube and add 25 ml of 1.0 mol/L ammonium acetate solution. Oscillate at 22±5°C, 220 rpm for 16 hours. Subsequent cleaning and transfer processes are the same as in step (1).

(4) residual fraction

Add 9.5 ml of prepared aqua regia (nitric acid: hydrochloric acid = 3:1) to the residue in the centrifuge tube after step (3), and let it sit at room temperature for 16 hours (overnight). Then, heat it in a water bath to a gentle boil and maintain for 2 hours. Due to the strong corrosive nature of aqua regia, it's important to strictly wear protective gear during this step, and the temperature should not be too high to prevent splashing and potential injury. After cooling to room temperature, proceed with the transfer process as in step (1).

Test S3 Determination of Cr(VI) Concentration

Determination of Cr^{6+} concentration referenced the national standard (GB7467-1987) using the diphenylcarbazide spectrophotometric method. The specific operational steps were as follows:

(1) Preparation of reagent I: Dissolve 0.02 g of diphenylcarbazide ($C_{13}H_{14}N_4O$) in 50 mL acetone, dilute with distilled water to 100 mL, mix well, and store the reagent in a brown bottle in the refrigerator for later use. If the color darkens, it should not be used.

(2) Take an appropriate amount of colorless and transparent water sample (hexavalent chromium content less than 50 μg) in a 10 mL centrifuge tube. Dilute it to 5 ml with distilled water, then add 0.5 mL of 5% sulfuric acid solution and 0.5 mL of 5% phosphoric acid solution, and mix well.

(3) Extract 2 mL of reagent I using a pipette and transfer it to a colorimetric tube, and mix well.

(4) Measure the absorbance of the aqueous solution using a UV-visible spectrophotometer. After 5-10 minutes, at a wavelength of 540 nm in a 10 mL colorimetric dish with distilled water as a reference, subtract the absorbance of the blank experiment to obtain the absorbance corresponding to Cr^{6+} in the water sample. Then, calculate the Cr^{6+} content based on the standard curve.

Table S1. Cr Content in Various Fractions of Soil Profile Layers.

The profile soil Cr speciation data. a, b, c represent soils containing 100 mg/kg, 200 mg/kg, and 300 mg/kg of chromium, respectively.

Rainwater pH.	Profile layers	F1	F2	F3	F4
7	a-1	6.347674	6.73649	42.83708	102.4588
	a-2	6.376004	4.861047	41.70879	69.31891
	a-3	6.004831	4.86577	45.49127	62.72571
	a-4	6.963894	4.631576	41.08649	76.36788
	a-5	6.823657	4.555853	42.92985	66.69329
	b-1	7.887957	10.37412	93.86023	64.63424
	b-2	9.438288	6.725194	86.029	91.32871
	b-3	9.608125	10.1359	93.95072	127.6943
	b-4	13.00397	9.827041	143.4462	109.6

	b-5	12.31633	6.942061	143.4174	121.8273
	c-1	12.06629	9.999097	141.529	78.56144
	c-2	12.25884	9.616079	127.4364	111.1001
	c-3	12.75708	6.918898	135.4953	114.1862
	c-4	10.84375	4.787442	91.98457	126.9003
	c-5	10.45702	4.373922	88.57414	71.60991
5	a-1	6.920871	7.204135	42.66532	70.16862
	a-2	7.434136	4.645221	45.0314	68.84754
	a-3	8.098474	8.012642	45.44752	75.49274
	a-4	6.556741	10.33232	45.41857	69.34283
	a-5	7.259938	4.78865	42.77961	78.02552
	b-1	10.7553	4.709949	91.49477	64.12484
	b-2	9.769724	7.091814	92.35603	94.82701
	b-3	11.09347	10.15358	100.1816	73.80518
	b-4	10.06257	9.854318	90.44974	61.81047
	b-5	9.781596	10.39482	89.06491	114.2642
	c-1	12.28725	10.66055	129.4012	109.6184
	c-2	12.04865	10.24638	132.4174	105.9471
	c-3	14.10051	12.96465	140.121	122.4179
	c-4	13.67325	6.737726	143.5736	105.5127
	c-5	13.45283	5.106059	141.6569	111.5568
3	a-1	5.840222	9.788805	39.19207	90.57938
	a-2	11.52634	6.992038	41.45401	65.32586
	a-3	13.45369	9.201854	40.27327	76.63652
	a-4	6.386292	10.07687	41.66817	65.00233
	a-5	6.7765	9.55646	40.38626	65.24078
	b-1	11.32874	6.745282	138.4835	63.49829
	b-2	15.35799	9.836022	139.3179	96.91908
	b-3	15.04418	6.379932	153.0516	112.5283
	b-4	14.00706	9.871142	141.2686	159.8438
3	b-5	13.16386	10.14427	142.6017	140.935
	c-1	6.832288	4.150697	126.932	130.9996
	c-2	14.03546	4.322409	135.8367	110.3711
	c-3	14.59083	9.677899	135.8483	135.3705
	c-4	14.3781	7.54654	140.0225	113.4663
	c-5	13.15597	4.622744	142.4912	127.7429

Table S2. Concentrations and Percentages of Cr(VI) and Cr(III) in the Leachate.

Number	Concentration of Cr(VI) (μg/L)	Percentage of Cr(VI) (%)	Concentration of Cr(III) (μg/L)	Percentage of Cr(III) (%)
1	196.80	34.63	371.53	65.37
2	139.32	29.04	340.41	70.96
3	189.83	45.34	228.87	54.66
4	148.03	45.20	179.48	54.80
5	141.07	44.62	175.09	55.38
6	128.87	45.70	153.11	54.30
7	121.91	52.54	110.14	47.46
8	106.23	50.58	103.81	49.42
9	99.27	50.76	96.28	49.24
10	97.53	48.48	103.66	51.52
11	78.37	40.26	116.30	59.74
12	81.85	47.65	89.93	52.35
13	76.63	43.97	97.64	56.03
14	73.15	45.78	86.61	54.22
15	66.18	44.78	81.62	55.22
16	45.28	42.89	60.30	57.11
17	38.31	42.60	51.63	57.40
18	27.86	36.64	48.19	63.36

19	24.38	37.22	41.13	62.78
20	26.12	40.32	38.66	59.68
21	22.64	42.32	30.85	57.68
22	20.90	41.20	29.82	58.80
23	24.38	46.02	28.60	53.98
24	20.90	36.47	36.41	63.53
25	19.16	33.91	37.34	66.09
26	15.67	28.41	39.49	71.59
27	19.16	40.33	28.34	59.67
28	19.16	39.12	29.81	60.88
29	17.42	37.39	29.16	62.61
30	13.93	34.19	26.82	65.81
31	1.74	9.93	15.80	90.07
32	1.74	9.37	16.84	90.63
33	1.74	12.62	12.05	87.38
34	1.74	14.49	10.28	85.51
35	1.74	12.77	11.89	87.23
36	1.74	14.80	10.02	85.20
37	1.74	11.51	13.38	88.49
38	1.74	11.14	13.89	88.86
39	1.74	12.09	12.67	87.91
40	8.71	74.70	2.95	25.30
41	1.74	10.81	14.37	89.19
42	1.74	14.77	10.05	85.23
43	1.74	14.50	10.27	85.50
44	1.74	16.14	9.05	83.86
45	1.74	17.23	8.37	82.77
